# Sustainment of proactive physical therapy for individuals with early-stage Parkinson’s disease: a quality improvement study over 4 years

**DOI:** 10.1186/s43058-021-00205-x

**Published:** 2021-09-27

**Authors:** Jillian MacDonald, Laura Doyle, Jennifer L. Moore, Miriam R. Rafferty

**Affiliations:** 1 Shirley Ryan AbilityLab, 355 E Erie St, 19th Floor AbilityLab, Chicago, IL 60611 USA; 2 Southestern Norway Regional Center of Knowledge Translation in Rehabilitation, Sunnaas Rehabilitation Hospital, Oslo, Norway; 3 Institute for Knowledge Translation, Carmel, IN USA; 4 Department of Physical Medicine and Rehabilitation, Feinberg School of Medicine Northwestern University, Chicago, IL USA; 5 Department of Psychiatry and Behavioral Science, Feinberg School of Medicine Northwestern University, Chicago, IL USA

**Keywords:** Sustainment, Spread, Implementation science, Parkinson’s disease, Physical therapy, Quality improvement

## Abstract

**Background:**

Implementation science frameworks aided the development of a new, evidence-based clinical physical therapy program. The purpose of this report is to describe the process of sustaining a clinical program in practice for over 4 years. We present a framework for integrating tools for sustainability with the Knowledge-to-Action model in the context of a proactive physical therapy (PAPT) program for individuals with early-stage Parkinson’s disease.

**Methods:**

Sustainability of implementation strategies was addressed using the Dynamic Sustainability Framework and sustainability assessment tools. Repeated retrospective medical record reviews and phone interviews were used to evaluate the reach and adoption of the PAPT over 4 years. Characteristics of those who engaged with PAPT, implementation fidelity, and clinical effectiveness were assessed for year 1 and year 3. Sustainability was measured using RE-AIM, NHS Sustainability Model, and Clinical Sustainability Assessment Tool.

**Results:**

Reach increased from 28 to 110 total patients per year and spread occurred from one to three sites. PAPT user age, sex, Hoehn and Yahr rating, time since diagnosis, and type of insurance were similar in year 1 and year 3 (*p* > 0.05). The program sustained its effect to help participants increase or maintain self-reported exercise (Y1, 95%; Y3, 100%). However, upon evaluation PAPT users in year 3 had longer time since symptom onset and worse UPDRS motor scores compared to the PAPT users in year 1 (*p* < 0.05). All sites sustained the core intervention components, with sustainability scores of 71/100 (± 9.9) on the NHS Sustainability Model and 6.1/7 (± 0.9) on the Clinical Sustainability Assessment Tool.

**Conclusions:**

Integrating multiple sustainability models and assessments supported continued effectiveness, spread, and sustainment of PAPT for 4 years. Effective planning, anticipating common healthcare changes, and addressing sustainability determinants early in program implementation were essential aspects of long-term success.

**Supplementary Information:**

The online version contains supplementary material available at 10.1186/s43058-021-00205-x.

Contribution to the literature

- Defining sustainability goals early in the implementation process, applying implementation frameworks, and using sustainability assessments may increase the maintenance of a novel evidence-based, clinical program.
- Repeating implementation evaluation frameworks, such as RE-AIM, can improve understanding of the clinical programs’ sustainment outcomes.
- A successful, evidenced-based proactive physical therapy program for individuals with early-stage PD can be sustained with careful attention to processes, staff, and organizational priorities.

## Introduction

Clinical practice guidelines support physical therapy (PT) in early Parkinson’s disease (PD) [1]. Despite these guidelines, payer datasets and patient registries reveal infrequent utilization of PT, particularly early after diagnosis [2, 3]. The proactive physical therapy (PAPT) program was established in 2016 to provide PT evaluations and individually tailored recommendations for physical activity and exercise in people with early PD [4]. The care path consists of neurologist referral to PT soon after diagnosis for a consultative model of PT. The patient receives one to four visits approximately every 6 months to monitor for changes and update PD-specific exercise prescription. The program improved access to PT for individuals with early PD and demonstrated effectiveness through self-reported increases in exercise and high satisfaction levels [4].

Established implementation frameworks informed the development of PAPT. The Knowledge-to-Action (KTA) Cycle was used to organize the process of implementation [5]. The Consolidated Framework for Implementation Research identified the implementation determinants [6]. The RE-AIM framework organized the initial program evaluation [7]. At the end of the first year, post-participation interviews suggested a demand for regional spread and sustainment to improve the program [4].

Sustainment, an essential component of implementation, has been described as using methods to preserve fidelity in an ever-changing healthcare environment [5, 7, 8]. Dynamic and adaptable implementation strategies increase the likelihood of sustained program fidelity [5, 8, 9]. Sustainment of a clinical program can occur in a single context or in combination with scaling or spread to new contexts, with consideration of environment-specific variations [10]. Recently, a definition of sustainability has emerged as “[1] after a defined period of time, [2] a program, clinical intervention, and/or implementation strategies continue to be delivered and/or [3] individual behavior change (i.e., clinician, patient) is maintained; [4] the program and individual behavior change may evolve or adapt while [5] continuing to produce benefits for individuals/systems” [11]. Several sustainability assessments for healthcare settings, including the National Health System (NHS) Sustainability Model and Clinical Sustainability Assessment Tool (CSAT) have been presented and are recommended to be performed repeatedly, but lack research on their application [12, 13].

This report describes the sustainment of PAPT as it spread to additional clinical sites over 4 years. We examine how integrating sustainability frameworks and clinical sustainability assessments supported sustainability.

## Methods

### Context

**Fig. 1:**
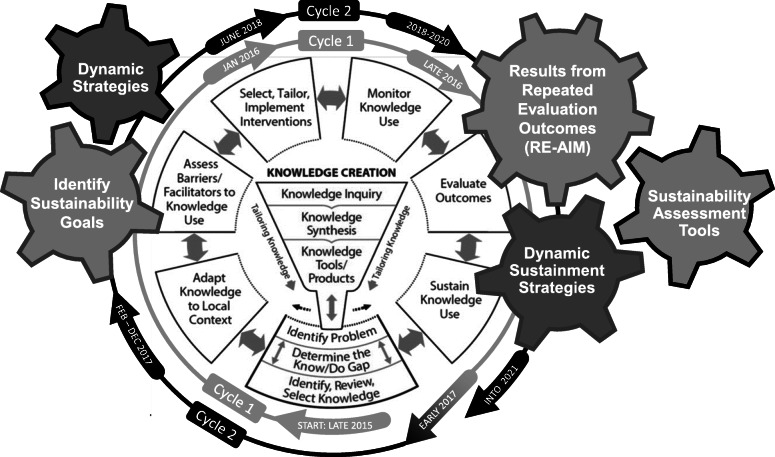
Applying sustainability frameworks during the implementation process. Sustainability frameworks and concepts are superimposed on the Knowledge-to-Action Cycle implementation process model (Reprinted with permission of John Wiley & Sons from: Straus SE, Tetroe J, Graham ID, eds. Knowledge Translation in Health Care: Moving From Evidence to Practice. 2nd ed. Chichester, United Kingdom: John Wiley & Sons Ltd; 2013.). The cogs have colored outlines to represent whether they were used in both (gray) or just the second sustainability cycle (black). Use of dynamic strategies assists with tailoring implementation strategies for sustainability. Using and then repeating implementation evaluation frameworks (e.g., RE-AIM), provides insight into sustainability outcomes. Sustainability assessment tools offer information about factors that affect sustainment and can guide further program adaptations

Proactive physical therapy was initially adopted at one urban, outpatient department within a large healthcare organization, designated as a Parkinson’s Center of Excellence [4]. PAPT requires changing processes to allow for early referral to PT with a consultative model. Expert clinicians receive training, particularly related to processes, but the provision of this guideline concordant care is aligned with best practice [1]. Rafferty and colleagues further describe the evidence-based clinical intervention and initial implementation [4]. Figure 1 provides a timeline showing a transition to sustainability-focused processes at the end of year one and integration of sustainability frameworks with implementation processes. Table 1 provides further definitions and description of how the implementation and sustainability frameworks were integrated to support the sustainment of PAPT. Spread occurred to two smaller suburban sites within the same rehabilitation system of care. The team leading the efforts included one physical therapist researcher, one clinical expert serving as a facilitator, the department managers, a physical therapist from the organization’s staff development program, and an external knowledge translation mentor.

**Table 1:** Putting frameworks into action: a summary of how implementation science frameworks are combined across the lifespan of an implementation project

Framework/concept	Description of framework and how it informs sustainment	Use in proactive physical therapy (PAPT)
Knowledge-to-action cycle (Graham 2006) [5]	Implementation process model. Sustainment phase to provide feedback for future cycles.	Implemented in Y1. Cycled through again in Y2–4.
Dynamic Sustainability Framework (Chambers, 2013) [9]	Emphasizes the importance of using dynamic strategies by describing the key tenets of sustainability within a changing delivery system. ∙ Ongoing intervention optimization ∙ Continuous intervention improvements for stakeholder learning ∙ Ongoing feedback on progress ∙ Organizational fit ∙ Voltage drop is not inevitable ∙ Organizational learning should be a core value ∙ Ongoing stakeholder involvement	∙ Ongoing intervention improvements supported through monthly meetings with bidirectional feedback, sharing new knowledge, organizational data and continuously updating resources. ∙ Organizational fit is managed through leadership engagement and institutionalization of practices. ∙ Organization leadership and facilitator support ongoing training and stakeholder engagement. ∙ Clinic culture promotes communication, engagement, and demand for increasing reach.
RE-AIM (Glasgow et al., 2019) [7] (www.re-aim.org)	An evaluative framework which promotes capture of outcomes from the five important dimensions: that consider internal and external validity in translational research. These dimension include: **reach** of the program, **effectiveness** of the implementation, **adoption** by the clinical team, **implementation fidelity**, and **maintenance** of the program. Maintenance specifically refers to when a program becomes “part of routine practices”.	∙ RE-AIM organized results based on the first iteration (Rafferty et al., [4]) and provided a useful comparison for sustainment outcomes of PAPT. ∙ In the first iteration, maintenance was considered broadly including both program and individual maintenance. ∙ In the second iterations, we added measureable sustainability goals and use of sustainability assessment tools.
Clinical Sustainability Assessment Tool (CSAT) (Luke et al, 2018; www.sustaintool.org) [13]	A tool to evaluate clinical program capacity for sustainment that assesses the following domains: engaged staff, engaged stakeholders, monitoring and evaluation, planning and implementation, outcomes and effectiveness, workflow integration, and organizational context by surveying clinical staff and stakeholders. **Psychometrics are not available currently**.	∙ Key stakeholders (*n* = 10) provided their insights into 7 key areas for sustainment in a survey at the end of the data collection period. ∙ Results of these assessments were shared back at the monthly meetings in order to address areas with lower ratings.
NHS Sustainability Model	This “diagnostic tool” was created to give insights to an implementation team on specific strengths and weaknesses of a current program and to help predict the likelihood of sustainment. The tool assesses processes, organization, and staff. **Psychometrics are not available currently**.	∙ Site champions and facilitator (*n* = 4) provided insights into sustainment of process, staff, and at the organizational level. ∙ Results of these assessments were shared back at the monthly meetings in order to address areas with lower ratings.

### Implementation and sustainability intervention

Implementation and sustainability strategies were identified through meetings with various stakeholders including therapists and leadership and further refined in an iterative process through ongoing monthly stakeholder meetings with clinician champions. As initial implementation transitioned to sustainment phases at the end of the first cycle through the Knowledge-to-Action Process (Fig. 1), we identified initial sustainability strategies. The Expert Recommendations for Implementing Change taxonomy was used to organize these strategies [14]. Strategies for spread and sustainment included a program facilitator to coordinate stakeholder communication, clinical site champions to facilitate local implementation as the program spread, organizational integration to maintain training resources across sites, and monthly clinical champion meetings for monitoring program barriers and facilitators, delivering feedback, and providing informal support. We applied the Dynamic Sustainability Framework (DSF) to help identify our need to adapt implementation strategies targeting the intervention, context, and ecological system [9]. Table 2 describes three examples of adaptations to sustainment strategies supported by the DSF. Other site-specific adaptations at the suburban clinics were primarily related to staff and processes, including [1] building and maintaining relationships with clients and referral sources for scheduling and long-term adherence, [2] staff support of clinical processes and paperwork, [3] leadership support strategies, [4] clinician training, and [5] providing interactive evaluative strategies to ensure fidelity. A complete list of the initial implementation strategies (2015–2016) and our sustainment strategies (2017–2019) are included in supplemental materials [Additional file 1]. While this program evaluation goes through 2019, the program continues to be sustained through 2021, with additional adaptations required to adjust to the COVID19 pandemic.

**Table 2:** Examples of adaptations for spread and sustainment. Adaptations were driven by consideration of the Dynamic Sustainability Framework (Chambers et al, [9])

**Adaptation for Intervention Spread and Sustainment**: • Each site had different processes for tracking patients for follow-up visits. • Technology issues prevented a clinician from editing a shared team document to support routine follow-up with PAPT users. • The clinician created a new system to track, provide reminders and communicate with patients. • Their system informed improvements the original system to support tracking patients after discussion at a monthly team meeting. *Take away*: • *Providing autonomy to champions can improve initial implementation processes*. • *Regular team meetings allowed for this process improvement to occur*. **Adaptation for Context (Practice Setting) Spread and Sustainment**: • Specific example: Staff turnover is a common problem that can stall implementation, spread, and sustainment. • One new champion prioritized educating their colleagues about the program in an inservice and identified another therapist for a succession plan in case of a staffing change. *Take away*: • *Staffing changes make continued program growth vulnerable*. • *Local opinion leaders and champions can help in addressing this by using the* “*train the trainer*” *method*. **Adaptation for Ecologic System (Outer Setting) Spread and Sustainment**: • A global pandemic resulted in changes in the delivery of care included clinic closure and transition to telehealth. • The proactive physical therapy delivery pattern was performed entirely in-person prior to the pandemic, but was adapted to telehealth within 1 month of the outpatient therapy clinic closing to in-person care. • The clinicians were able to use monthly meeting times to discuss the modifications needed for a rapid and safe transition to telehealth. *Take away*: • *A climate where clinicians felt supported and confident to suggest changes can accelerate adaptations to unpredictable challenges*.	

### Study of intervention

#### Data sources and participants

Data sources include [1] administrative data from the outpatient department on referrals and utilization (2016–2019), [2] retrospective electronic medical records (EMR) of first-time PAPT users in year 1 (Y1) (2016) and year 3 (Y3) (2018), [3] structured interview responses conducted over the phone with first-time PAPT users from Y1 and Y3 who responded to a quality improvement phone interview request, and [4] stakeholder survey results from the NHS Sustainability Model and CSAT to evaluate sustainment after completion of year 4 (Y4). PAPT users include those who were referred and attended the PAPT program. Eligible PAPT users were referred with a mild or moderate PD diagnosis or suspected prodromal PD (e.g., REM sleep behavior disorder, hyposmia) who accessed the program for the first time in Y1 and Y3. PAPT users opted in to interviews after they were sent a letter and screening phone call. Three researchers (JM, AS, EN) reviewed information from the interviews. Participants were excluded from this analysis if they were referred to the PAPT program with a different diagnosis. A champion at each site (*n* = 3) and one program facilitator were invited to complete the NHS Sustainability Model and the CSAT. Additionally, five organization leaders and three referrers were invited to complete the CSAT.

#### Measures

The RE-AIM framework was used to assess and describe the spread and sustainment outcomes of PAPT, comparing outcomes from Y1 and Y3 or Y4. *Reach* of the program was measured by providing the number of PAPT users across all sites, the number of first-time users, and the number retained each year (i.e., an individual who accessed PAPT in a previous year). *Effectiveness* and satisfaction were evaluated with quality improvement phone interviews. Questions included (1) PAPT user self-reported changes in exercise, (2) self-reported benefit of PAPT on an 11 point scale from 0 (not beneficial) to 10 (extremely beneficial), and (3) recommendation to others on a similar scale from 0 (not likely to recommend at all) to 10 (extremely likely to recommend). *Adoption* was measured as the (1) number of sites, (2) number of physical therapists trained and using the PAPT care model, (3) referral numbers from targeted referrers, (4) proportion of PAPT users who attended versus the number referred to PAPT, and (5) number of PAPT users who engaged in a long-term follow-up episode of care. *Implementation fidelity* was assessed by EMR review of (1) physical activity and exercise prescription for PAPT users through documented home exercise prescription and (2) PAPT care path utilization.

*Maintenance* was assessed through sustainability assessments that were administered after year four. The NHS Sustainability Model includes ten questions in three domains: staff, organization, and processes. Each item is measured on a 4-point categorical scale and summed into a total score with a maximum of 100 points. This model’s training materials propose that a score over 55 indicates optimism toward sustaining the program [12]. The CSAT contains 35 questions in seven domains: engaged staff and leaders, engaged stakeholders, organizational readiness, workflow integration, implementation and training, monitoring and evaluation, and outcomes and effectiveness. Each item is scored on a 7-point response scale, which is averaged within each domain and across domains for a total score from 1 to 7 [13].

#### Analysis

Data are presented with descriptive statistics. PAPT user characteristics, interview responses, and documentation of information related to program fidelity from Y1 and Y3 were compared with an unpaired *t*-test or Chi-square test based on the type of data.

#### Ethical considerations

Because of the nature of quality improvement and program evaluation the Northwestern University Institutional Review Board determined that this EMR data extraction and structured interviews did not qualify as human subjects research. Data from all sources were collected and maintained using HIPAA compliant methods. Reporting was completed using the Standards for Quality Improvement Reporting Excellence (Additional file 2) [15].

## Results

The results of program spread and sustainment are presented in Table 3 using the RE-AIM framework. Sustained PAPT is measured in years 3 and 4.

**Table 3:** Program results presented with RE-AIM in the initial year and sustained over 3–4 years

	First year of PAPT	Sustained PAPT
**Reach**		
Access to PAPT	28 PAPT users	110 total PAPT users^b^ 84 new PAPT users^b^
**Effectiveness (program)**		
Program benefit (median)	9/10 from survey respondents	9/10 from survey respondents^a^
**Effectiveness (clinical)**		
Self-report increase in exercise	70%	64%^a^
**Adoption**		
Clinics	1 clinic	3 clinics^b^
Total therapists	2 PTs	10 PTs trained over the project 6 PTs in practice^b^
Total referrers	4	15^b^
**Implementation fidelity**		
PAPT carepath use	86%	84%^a^
Aerobic exercise instruction	79%	87% ^a^
**Maintenance**		
Average NHS Sustainability Model score	Not applicable	71/100 (range = 57.3–81)^b^
Average Clinical Sustainability Assessment Tool score	Not applicable	6.1/7 (range = 5.7–6.5)^b^

^a^Year 3

^b^Year 4

**Fig. 2:**
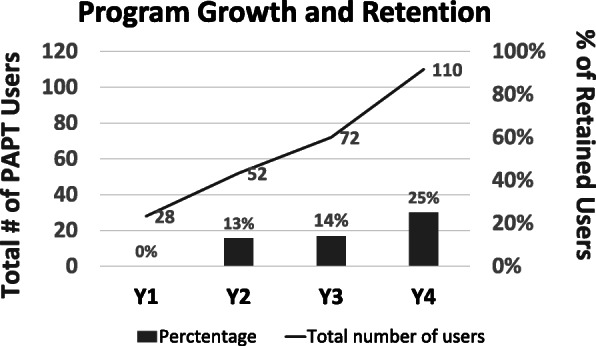
PAPT Program growth and retention across 4 years

The PAPT program’s *reach* to new users more than doubled (28 to 62) over the first 4 years and is presented in Fig. 2. Comparing demographics between Y1 and Y3, the PAPT users have similar age, sex, Hoehn and Yahr stage of PD, time since diagnosis, and insurance [Table 4]. However, in Y3, the program reached individuals with a worse Unified Parkinson’s Disease Rating Scale (UPDRS) motor disease severity score and longer time since symptom onset.

**Table 4:** Demographics and clinical characteristics of proactive physical therapy users in years 1 and 3

	Year 1 (*n* = 28)	Year 3 (*n* = 62)	*t*-value or *X*^2^ (DF)	*p-*value^a^
Age [mean years (SD)]	65.1 (11.2)	65.8 (9.1)	*t* (88 DF) = − 0.30,	*p* = 0.761
Male [*n* (%)]	16 (57%)	37 (59%)	*X*^2^ (1 DF) = 0.05	*p* = 0.821
UPDRS [mean (SD)]	16.2 (6.8)	23.2 (12.0)	*t* (50.7 DF) = − 2.78	*p* = 0.0076
*Missing*^*a*^	0	30		
Hoehn and Yahr stage			*X*^2^ (2 DF) = 1.75	*p* = 0.418
*1*	5 (18%)	9 (15%)		
*2*	23 (82%)	23 (37%)		
*3*	0 (0%)	1 (2%)		
*Missing*^*a*^	0 (%)	29 (47%)		
Time since diagnosis			*X*^2^ (4 DF) = 9.25	*p* = 0.055
*< 1 month*	14 (50%)	12 (19%)		
*1–6 months*	4 (14%)	19 (31%)		
*6-12 months*	4 (14%)	8 (13%)		
*1–2 years*	3 (11%)	7 (11%)		
*2+ years*	3 (11%)	13 (21%)		
*Missing* ^*a*^	0 (0%)	3 (5%)		
Time since symptoms			*X*^2^ (3 DF) = 9.06	*p* = 0.029
*< 1 month*	0 (0%)	0 (0%)		
*1–6 months*	5 (18%)	1 (2%)		
*6–12 months*	5 (18%)	11 (18%)		
*1–2 years*	5 (18 %)	20 (32%)		
*2+ years*	13 (46%)	23 (37%)		
*Missing* ^*a*^	0 (0%)	7 (11%)		
Insurance			*X*^2^ (2 DF) = 1.14	*p* = 0.565
*Medicare*	15 (54%)	29 (47%)		
*Blue Cross Blue Shield*	9 (32%)	18 (29%)		
*Other*	4 (14%)	15 (26%)		

^a^Missing data were excluded from analyses (UPDRS and Hoehn and Yahr were missing from provider referral notes)

**Fig. 3:**
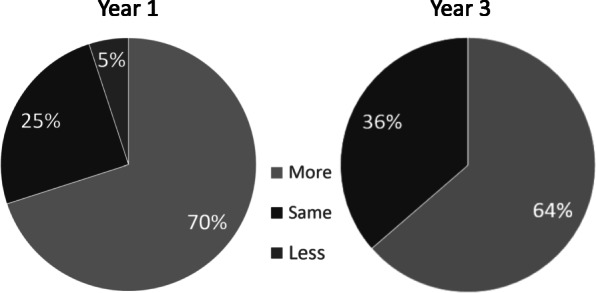
PAPT Program Effectiveness in year 1 and year 3. Report of amount of exercise in the year after accessing PAPT

Quality improvement phone calls measuring sustained program *effectiveness* were completed in 71% of 28 users in Y1 and 53% of 62 new users in Y3 (*χ*^2^_df=2_ = 2.64, *p* = 0.104). The proportion of individuals in Y1 and Y3 with self-reported increased or maintained exercise is presented in Table 3 and was found to be not different between the years studied (Fig. 3; *χ*^2^_df=2_ = 0.228, *p* = 0.3283).There was increased *adoption* by outpatient clinics, physical therapists, and referrers (Table 3). Furthermore, the four initial referrers increased their overall PT referrals (PAPT program and other PT) from 115 referrals in the 2016 fiscal year to 167 in the 2019 fiscal year, a 45% increase. In the same period, the referring clinic grew approximately 11% year-over-year (approximately 37% over 4 years), suggesting that PT referrals increased beyond volume-based growth. At the level of the PAPT user, the proportion of referred individuals who completed a PAPT evaluation improved from 74% in Y1 to 84% in Y4. Finally, 37 of all 135 unique PAPT users (27%) have adopted the long-term follow-up model, defined as attending at least one return episode.

*Implementation fidelity*, assessed by the proportion of PAPT users who required just 1–4 visits in accordance with the original PAPT care path, was similar in Y1 and Y3 (*χ*^2^_df=2_ = 0.55, *p* = 0.760). Clinicians also documented explicit aerobic exercise prescription during the initial PAPT visit similarly in Y1 and Y3 (*χ*^2^_df=1_ = 1.07, *p* = 0.302).

Program *maintenance* was measured using two sustainability assessments. All stakeholders who were sent a request for the NHS Sustainability Model completed the survey (*n* = 4) and all but two referrers completed the CSAT (*n* = 10). The average NHS Sustainability score 71 (out of a possible 100 points) and the average CSAT score was 6.1 (out of a possible 7). Specific category scores are presented in Table 5. Perfect scores were reported in two NHS sustainability factors: “clinical leadership engagement and support” and “fit with the organizational culture.”

**Table 5:** Sustainability scores from stakeholders using the NHS Sustainability Model and Clinical Sustainability Assessment Tool (CSAT)

Scale and factor/domain	Average rating (range)
**Staff and stakeholders**	
NHS: clinical leadership engagement	15/15
NHS: staff involvement and training to sustain the process	8.6/11.4 (0–11.4)
NHS: senior leadership engagement	8.2/15 (5.7–15)
NHS: staff behaviors toward sustaining the change	6.6/11 (5.1–11)
CSAT: engaged staff and leadership	6.5/7 (6.0–7.0)
CSAT: engaged stakeholders	6.3/7 (5.6–7.0)
**Organization**	
NHS: fit with the organization's strategic aims and culture	7/7
NHS: infrastructure for sustainability	4.5/9.5 (0–9.5)
CSAT: organizational readiness	5.9/7 (4.6–6.6)
**Process and outcomes**	
NHS: credibility of the benefits	7.7/9.1 (6.3–9.1)
NHS: effectiveness of the system to monitor progress	4.7/6.5 (2.4–6.5)
NHS: adaptability of improved process	4.3/7 (3.4–7)
NHS: benefits beyond helping patients	4.5/8.5 (0–8.5)
CSAT: outcomes and Effectiveness	6.5/7 (5.8–7.0)
CSAT: implementation and training	6.0/7 (4.8–7.0)
CSAT: workflow integration	5.9/7 (4.4–7.0)
CSAT: monitoring and evaluation	5.7/7 (4.4–7.0)

## Discussion

Attention to sustainability frameworks has facilitated PAPT program spread to three sites within one healthcare organization and long-term sustainment. In Fig. 1, we depict the integration of sustainability tools with our implementation process, determinants, and outcomes frameworks to provide one example of how to reconcile the challenges to long-term program sustainment [9, 12, 13, 16]. The effort to identify dynamic, adaptable implementation strategies early in the implementation process using the DSF helped prepare for success despite typical and atypical healthcare setting changes (e.g., staffing changes, technology challenges, transition to telehealth) [9]. Additional insights into how our processes, staff, and organization promoted adaptation and addressed these challenges, see Table 2. Although formal sustainability assessments were not completed until year four in this project, repetition of evaluative tools is recommended (e.g., RE-AIM) [17] and using sustainability assessments (e.g. CSAT and the NHS Sustainability Model) to promote a program’s sustained effectiveness (Fig. 1).

Use of existing sustainability frameworks aids in identifying and addressing sustainability barriers. Changing barriers over time could result in “program drift,” or deviation from the program’s original aims [9]. The DSF promotes the maintenance of high-fidelity interventions through identification of adaptable implementation strategies [9]. In this study, a facilitator worked closely with trained, clinical champions at each site who monitored and led program adaptations. The champion meetings, continued adaptations of clinical tools, and electronic resources for PAPT users were sustained through healthcare staffing changes (including champion transitions), as well as less common changes such as moving facilities and a pandemic. At one clinic, the champion had autonomy to create a tracking system to address a process sustainability barrier. At the other site, the champion trained an additional therapist and educated the clinical team due to staff sustainability barriers. Use of evaluative sustainment frameworks and assessments enabled the appraisal of ongoing implementation strategies and further tailor to site-specific needs. After administering the CSAT and the NHS Sustainability Model, the facilitator could assist the sites to identify adaptations to meet their site-specific sustainability barriers.

This program evaluation uniquely focuses on sustainability of a clinical program targeting an outpatient physical rehabilitation setting. In two recent reviews on program sustainability, none occurred in similar departments [16, 18]. However, in one quasi-experimental study, behavior change strategies were implemented in a PT clinic with good uptake during the intervention, but poor sustainment at the 3-, 6-, or 12-month follow-up [19]. Our sustained implementation strategies using the EMR, centralizing education, and programmatic support of a facilitator and champions have helped this program to be successful for more than 4 years.

Following initial implementation, demand from stakeholders, including referrers, patients, therapists, and managers, led to the spread of PAPT through our regional system of care. PAPT program growth was associated with minor population changes in the PAPT program (Table 3), and increased PT referrals from the initial referrers, which may help to address the PT referral and utilization gap noted in the health services literature [2]. Adoption at new sites was accompanied by site-specific processes and implementation strategies that have been essential for the success in unique contexts.

Unintentional spread also occurred through adoption of the proactive, consultative care model by speech, and occupational therapy for people with PD, as well as for other patient diagnoses, like Huntington’s disease. Data from these clinical areas were excluded from this analysis but are well supported in the literature and bolstered PAPT sustainability [1, 20–22]. Organization leaders were empowered to lead these new programs by adapting the implementation strategies associated with the success of PAPT.

Two limitations include the lack of generalizability of retrospective quality improvement data and lack of well-studied sustainability measures. The use of retrospective measurement in a single health system limits generalizability to other organizations. However, documenting strategies to spread from academic to suburban clinics in the same health system may be impactful in similar contexts. The retrospective methods also present challenges such as inability to control potential confounding factors, including new leadership positions within the organization. The second limitation is that the existing clinical sustainability measures have limited reliability and validity data [12, 13]. Implementation practitioners should consider using new sustainability tools as they are validated; however the varied contexts of sustainability between communities and clinical settings may require measurement adaptations [23]. Despite their limitations, our clinical and research team felt that the chosen assessment tools provided valuable information for program sustainment.

Demonstrating the application of sustainability frameworks has implications for implementation practice and research. Figure 1 provides an example for integrating sustainability principles during implementation. Key features are identifying and addressing sustainment early, repeating implementation evaluations over time, and completing sustainability assessments. More research can improve our understanding of the complexities of sustainability. Systematic implementation research studies should measure the benefits of assessing and addressing sustainability at different implementation stages.

## Conclusions

We integrated sustainability into implementation frameworks to assist with creating a resilient program and to provide a tool to improve program development. Attention to sustainability when selecting adaptable implementation strategies, repeated evaluation, and sustainability assessments successfully assisted with the 4-year sustainment of the PAPT program.

## Supplementary Information

**Additional file 1.** Initial and sustained implementation strategies. Strategies are organized via Expert Recommendations for Implementing Change (ERIC) taxonomy (Waltz et al. 2015) Strategies bolded are those that continue within the organization. Strategies described in ERIC that were not applied are not included in this table.

**Additional file 2.** SQUIRE.

## Data Availability

The datasets generated by the current study are not publicly available but are available from the corresponding author on reasonable request.

## Abbreviations

PAPT: Proactive physical therapy
PT: Physical therapy
PD: Parkinson’s disease
KTA: Knowledge-to-Action (Cycle)
RE-AIM: Reach, Effectiveness, Adoption, Implementation Fidelity, Maintenance
CSAT: Clinical Sustainability Assessment Tool
DSF: Dynamic Sustainability Framework
EMR: Electronic Medical Record
Y1: Year 1
Y3: Year 3
Y4: Year 4
UPDRS: United Parkinson’s Disease Rating Scale
