# Supplementary material for: Sustainment of proactive physical therapy for individuals with early-stage Parkinson’s disease: a quality improvement study over 4 years
Source: Implement Sci Commun. 2021 Sep 27;2:111. doi: 10.1186/s43058-021-00205-x (PMC8477476; doi:10.1186/s43058-021-00205-x)
Supplement: Supplementary file 1 — Additional file 1. Initial and sustained implementation strategies. Strategies are organized via Expert Recommendations for Implementing Change (ERIC) taxonomy (Waltz et al. 2015) Strategies bolded are those that continue within the organization. Strategies described in ERIC that were not applied are not included in this table. [file 43058_2021_205_MOESM1_ESM.pdf]

Additional file 1: Initial and sustained implementation strategies, organized with the Expert Recommendations for Implementing Change (ERIC; Waltz et al 2019). Bolded sustainment strategies indicate those continued to present (2021).

Implementation Strategy	Initial Implementation Strategies (2015-2016)	Sustainment Strategies (2017-2019)
ERIC Cluster 1: Use evaluative and iterative strategies	Assess for readiness and identify barriers and facilitators	
	• Small group of stakeholders informally discussed their views	• Stakeholder meetings with entire clinical team at one site and interview off-site champions and managers<br>• Periodic assessment in monthly meetings with champions<br>• **Plan to continue gathering input from PDMD clinical champion meetings.**
	Audit and provide feedback	
	• Two therapists compared performance	• Provide one page document of feedback after initial three sessions with participant within 3-5 days.<br>• **Champions to perform three audits/year.**
	Purposefully reexamine the implementation	
	• Informally with communication between two therapists	• **Facilitator &/or program manager attend to feedback at champion meetings allowed for dynamic changes while maintaining the fidelity of the program.**<br>• **Utilization of Sustainability Assessment Tools with stakeholders to assess the program**
	Develop implement tools for quality monitoring	
	• Two therapists compared documentation of physical activity<br>• PAPT user survey<br>• Clinical outcomes manually reviewed	• PAPT user survey repeated<br>• **Clinical outcomes manually reviewed**<br>• **Development of documentation for PA for easy clinician review .**
	Develop and organize quality monitoring systems	
	• Two therapists develop survey for PAPT users	• Survey updated for PAPT users by clinicians<br>• **Program managers plans to use organizational survey data**
	Develop a formal implementation blueprint	
	• Care path developed	• Continued care path<br>• Grid created to aid in scaling to other disciplines & in the development of PAPT at additional sites.
	Conduct local need assessment	
	• Informal discussions with stakeholders	• Focus group, interviews with stakeholders.<br>• **Multiple stakeholder groups meet regularly, some institutional processes already in place (e.g. regular PDMD**

(continued) ERIC Cluster 1: Use evaluative and iterative strategies		**champions meet, leadership meetings meetings)**
	Obtain and use patients/consumers and family feedback.	
	• Interview performed with Year 1 PAPT users	• Interviews performed with those PAPT users seen during Year 3
ERIC Category 2: Provide interactive assistance	Facilitation	
	• Two therapists worked together	• Formal facilitator role created<br>• Program manager to continue to engage this population, work with champions
	Provide Local Technical Assistance	
	• Informal	• Informal
	Provide Clinical Supervision	
	• Limited supervision, mostly self- review	• Chart audit on first three sessions<br>• Periodic review or support when requested<br>• **Institutional mentorship programs to support clinicians**
	Centralize Technical Assistance	
	• Informal	• Informal
ERIC Cluster 3: Adapt and tailor to context	Tailor strategies	
	• Strategies tailored to some stakeholders	• Tailor strategies to multiple stakeholders, multiple sites with input from a variety of stakeholders<br>• Strategies to be updated by champions with input from program manager as needed.
	Promote adaptability	
	• With only two therapists, change could be discussed and implemented	• Feedback taken with initial interviews, monthly meetings.<br>• Regular communication allowed for a flexible time table based on clinic volumes<br>• Utilized electronically shared resources<br>• Review of patient outcomes, quality improvement interviews aid in process improvement.
	Use data experts	
	• Knowledge broker	• Knowledge broker
ERIC Cluster 4: Develop stakeholder interrelationships	Identify and prepare champions	
	• Managers identified therapist	• **Leadership identify champions**
	Organize clinician implementation team meetings	
	• Informal	• Monthly meetings set<br>• **Champion meetings**
	Recruit, designate, and train for leadership	
	• One PT identified by lead researcher & manager	• Team of three attend Knowledge Translation summit, other attend PD-related course<br>• **Plans champion orientation, webinars, and journal club participation**.
	Inform local opinion leaders	

(continued)<br>ERIC Cluster 4: Develop stakeholder interrelationships	• Interviews with key referrers, OP manager, therapist	• Interviews/focus groups before implementation, intermittent updates to team<br>• **PAPT updates included in physician leader's monthly updates.**
	Build a coalition	
	• Attended meetings with referrers	• Engaged stakeholders of the entire OP team<br>• **Network of champions at each site, engaged with leadership.**
	Obtain formal commitments	
	• Not applied	• **Champion role commitment**
	Identify early adopters	
	• Manager identified one staff	• **Managers and implementation identified champions**<br>• **Champions worked with managers to identify other therapists to be trained**
	Conduct local consensus discussions	
	• Discussions with referrers at collaborative meetings	• Focus groups with therapists, managers<br>• Quality Improvement interviews with PAPT users<br>• **Monthly meetings with champions**
	Capture and share local knowledge	
	• Not applied	• **Champions provide insight and contributions.**
	Use an implementation advisor	
	• Knowledge broker involved.	• Knowledge broker facilitates team attendance to Knowledge Translation Summit<br>• Support from knowledge translation mentors
	Visit other sites	
	• Not applied	• Planned, but not accomplished by our immediate team.<br>• **PD program manager visits to assess & support settings.**
	Develop an implementation glossary	
	• Not applied	• Informal, general protocol developed<br>• Dissemination toolbox
	Develop academic partnerships	
	• Researcher from academic center collaborates with clinical team	• **Collaboration with research & clinical team**<br>• **Team engaged with organization internal staff development.**
ERIC Cluster 5: Train and evaluate stakeholders	Train and educate stakeholders	
	• Referral development: Targeted academic medical center neurologists	• Referral Development: Developed new materials to target community neurologists<br>• **Share materials and highlight key statements for marketing material in dissemination toolbox**
	Provide ongoing consultation	

(continued) ERIC Cluster 5: Train and evaluate stakeholders	• Two therapists discussed regularly	• Knowledge broker, facilitator available for questions, attended meetings regularly<br>• **Champions meet regularly**<br>• **Access to mentoring**
	Develop educational materials	
	• Materials for PAPT Users: Shared decision making handout<br>• Materials for therapists: evaluation plan for therapist	• **Materials for PAPT Users: shared decision making handout (updated every two years), progression handouts**<br>• Material for therapists: presentation, **training resources**, **webinar for therapist**
	Make training dynamic	
	• One co-treatment session	• **Presentation converted to webinar**<br>• Shadowing<br>• Questions for therapists
	Distribute educational materials	
	• Two therapists shared materials	• Shared folder<br>• Tool box with important materials
	Use train-the-trainer strategies	
	• Two therapists collaborated	• **Trained champions train other staff at home site**
	Conduct educational meetings	
	• Informal	• Monthly PAPT meetings<br>• Planned champion meetings
	Conduct educational outreach visits	
	• Not applied	• **Presentations at affiliate hospital**
	Shadow other experts	
	• Two therapists co-treat	• Shadowing is part of the training<br>• Plan to continue to shadow with training, happening with OT & SLP teams
	Work with educational institutions	
	• Affiliate medical center team	• Knowledge Translation Summit<br>• Internal staff development, clinical programs at organization and affiliate medical center.
ERIC Cluster 6: Support Clinicians	Facilitate relay of clinical data to providers	
	• Clinicians assisted with quality improvement data collection	• Clinician champions worked on implementation materials, monthly meetings provided opportunity for information, clinicians presented data at national conference platform and poster<br>• **Champions and leaders will now do this.**
	Remind clinicians	
	• Informal	• **Monthly meetings provided an opportunity for champion discussion and reminders.**<br>•
	Develop resource sharing agreements	

(continued) ERIC Cluster 6: Support Clinicians	• Not applied	• Staff collaborated on resources (no formal agreements)
	Revise professional roles	
	• Not applied	• Manager role developed by the organization who acted as an advocate for the program.
	Create new clinical teams	
	• Not applied	• **Champion team**, clinical teams
ERIC Cluster 7: Engage consumers	Involve patients/consumers and family members	
	• Interviewed 2016 attendees for feedback	• Interviewed 2018 PAPT users and shared quality improvement feedback with clinicians<br>• **Informal reflections from trained clinicians at monthly meetings.**
	Intervene with patients/consumers to enhance uptake and adherence	
	• Evaluations & relationship with patients/consumers foster better adherence	• **Evaluations & relationship with patients/consumers**
	Prepare patients/consumers to be active participants	
	• Strong relationship	• **Strong relationship**
	Increased demand	
	• Educate referrers	• Marketing resources for individuals with PD, external referrers
ERIC Cluster 8: Utilize Financial Structures	Fund and contract for the clinical innovation	
	• Internal department funds<br>• Parkinson's Foundation Grant	• Grant funding through Academy of Neurologic Physical Therapy.<br>• General clinic specific organizational funds<br>• Marketing department<br>• Internal Academy
	Access new funding	
	• Parkinson's Foundation Grant	• Knowledge Translation Summit Grant
ERIC Cluster 9: Change Infastructure	Mandate change	
	• Not applied	• Applied with support from organizational leaders
	Change record systems	
	• Not applied	• **Outcome Measure added: Mini-BESTest**<br>• In process: Physical Activity
	Change service sites	
	• Not applied	• Three additional locations added to increase reach

*PDMD: Parkinson's disease and Movement Disorders, PAPT: ProActive Physical Therapy, Parkinson's disease (PD)
